# Supplementary material for: Affordability of Habitual (Unhealthy) and Recommended (Healthy) Diets in the Illawarra Using the Healthy Diets ASAP Protocol
Source: Int J Environ Res Public Health. 2025 May 13;22(5):768. doi: 10.3390/ijerph22050768 (PMC12110866; doi:10.3390/ijerph22050768)
Supplement: Supplementary file 1 [file ijerph-22-00768-s001.zip › ijerph-3522731-supplementary.pdf]

International Journal of
Environmental Research and Public Health

MDPI

Supplementary material

Table S1. Population demographics for each location included in the study.

SA2 location	Port Kembla-Warrawong [57]	Warilla [58]	Unanderra-Mount Kembla [59]	Balgownie-Fairy Meadow [60]	Thirroul-Austinmer-Coalcliff [61]	Helensburgh [62]
**SEIFA quintile**	1	1	3	3	5	5
**Median fortnightly household income ($AUD)**	2411	2592	4178	3558	5272	5531
**Median fortnightly personal income ($AUD)**	1208	1277	1660	1584	2206	2187
**Mean number of children per family with children.**	1.8	1.8	1.9	1.8	1.9	1.9
**Family households with children (%)**	23.5	23.0	36.9	28.7	38.4	46.6
**Single parent households (%)**	16.9	18.0	11.6	11.9	8.2	9.0
**Persons aged >70 (%)**	16.4	16.8	13.5	15.6	11.7	9.1
**Persons not in the labour force (%)**	47.7	44.7	37.2	38.1	33.9	27.9
**In the labour force: unemployed (%)**	7.3	6.4	4.2	4.5	3.8	3.1

Sourced from Australian Bureau of Statistics QuickStats [57-62]

Lower quintiles are associated with higher proportion of retirees, single parent families, persons not in the labour force, and unemployed persons. This impacts the median wage as many will be on welfare, which is well below the poverty line, or using superannuation, which is not considered an income.

.

Table S2. Quantities of food (not adjusted for edible/cooked portions) for each household and diet rounded to the nearest mL or g [12]

	**Family of four**		**Single parent family**		**Single male**	
**Food item**	R (g/mL)	H (g/mL)	R (g/mL)	H (g/mL)	R (g/mL)	H (g/mL)
**Water**						
Water, bottled	5296	5296	3275	3275	2021	2021
**FRUIT**						
Apples	5897	3777	4385	2996	1512	780
Bananas	8408	1384	6252	933	2156	451
Oranges	7098	2163	4060	1695	1820	468
Fruit salad in juice		3069		2137		932
**VEGETABLES AND LEGUMES**						
Broccoli	2455	705	1870	463	585	240
Carrot	2867	979	2184	758	682	221
Iceberg lettuce	1955	1057	1490	622	465	435
Onions	2047	105	1522	46	525	60
Potato	2738	1723	1912	1114	826	609
Pumpkin	2756	300	2100	193	656	107
Tomatoes	1654	492	1230	304	424	189
White cabbage	1837	294	1400	176	438	118
Baked Beans (canned)	1005	369	480	241	525	128
Diced tomatoes (canned)	1638	235	1218	141	420	94
Four bean mix (canned)	1005	74	480	50	525	24
Sweetcorn (canned)	1160	206	810	130	650	77
Mixed vegetables (frozen)	1638	1184	1218	760	420	424
Peas (frozen)	1638	273	1218	167	420	106
**Grain (Cereal) Foods – Wholegrain and Refined**						
Cornflakes	670	680	502	525	168	155
Rolled oats	6648	870	4968	615	1680	255
Weetbix	2216	430	1656	263	560	167
White bread, pre-packaged	893	3033	669	2344	224	689
Wholemeal bread, pre-packaged	4272	1054	3152	683	1120	370
White pasta	674	438	495	311	179	127
White rice	1021	811	750	549	271	262
Dry wheat crackers, water crackers	781	258	585	225	196	33
**Lean Meats and Poultry, Fish, Eggs, Nuts and Seeds**						
Beef mince, lean	1460	334	1081	196	379	139
Beef rump steak	1465	1320	1085	854	380	466
Lamb loin chops	2116	465	1567	150	548	315
Chicken, cooked whole	1133	1279	845	773	287	506
Tuna (canned in oil)	2209	1262	1649	907	560	356

Eggs	2517	994	1879	644	638	349
Peanuts, unsalted	780		360		420	
Canned meat and vegetable casserole		1291		864		427
**Milk, Yoghurt, Cheese and Alternatives**						
Milk (regular)	6438	5961	4938	4702	1500	1259
Milk (lite)	12000	2929	9000	2102	3000	828
Cheese (regular)	704	624	544	467	160	157
Cheese (reduced fat)	516	44	396	29	120	15
Plain Yoghurt (regular)	2576	204	1976	127	600	77
Yoghurt (reduced fat)	5100	676	3900	467	1200	209
**Unsaturated Oils and Spreads (or foods from which these are derived)**						
Canola margarine	412	170	226	127	186	43
vegetable oil	291	7	160	4	131	3
Olive oil	291	7	160	4	131	3
**Pre-prepared mixed foods**						
Sandwich, pre-made, white bread, chicken, salad, cheese	560	361	400	174	160	186
**Discretionary Choices**						
Beer, full strength		4661		428		4232
White wine, sparkling		863		572		291
Whisky		266		97		170
Red wine		1078		491		587
Orange fruit juice		6053		4734		1319
Artificially sweetened soft drink		2390		1419		972
Sugar-sweetened soft drink (Coca Cola)		12012		8612		3400
Flavoured milk		2416		1529		888
Butter		280		224		56
Salad dressing		227		187		90
Chocolate		441		333		108
Confectionary		418		373		46
Cream-filled sweet biscuit, pre-packaged		496		359		136
Ice cream		1830		1561		269
Instant noodles, wheat based		381		301		79
Muesli bar, pre-packaged		373		301		72
Muffin, packaged		1455		1104		352
Nuts, mixed, salted		255		154		102
Potato chips, pre-packaged		518		420		98
Potato chips (hot), commercial		670		446		225
Savoury flavoured biscuits		222		182		40

Chicken soup (canned)		1340		715		625
Hamburger, commercial		2413		1749		664
Lasagne, pre-packaged (frozen)		4322		3298		1024
Meat pie, commercial		1638		1194		444
Pizza, commercial		1182		872		310
Beef sausages		1047		701		346
Fish fillet crumbed, pre-packaged (frozen)		302		182		120
Ham		189		129		60
Tomato sauce		569		397		172
White sugar		566		373		194

Abbreviations: Habitual diet (H), Recommended diet (R), Millilitres (mL), grams (g)

List S1. Healthy Diets ASAP food price collection protocol [12]

1. Record the usual price of an item, i.e. do not collect the sale/special price unless it is the only price available (if so, note in comment column).
2. Look for the specified brand and specified size for each food item and record the price.
   - If the specified brand is not available: Choose the cheapest brand (non-generic) available in the specified size. Note this brand in the "Your brand" column.
   - If the specified size is not available: Choose the nearest larger size in the specified brand. If a larger size is not available, choose the nearest smaller size. Note this size in the "Your size" column.
   - If both the specified brand and specified size are not available: Choose the cheapest in the nearest larger size of another brand (non-generic). If a larger size is not available, choose the nearest smaller size.
   - If multiple brands are specified, record the price of the cheapest one and note brand in the "Your brand" column.
   - If the item is only available in a generic form (e.g. Home Brand, Coles, Woolworths Select, Black and Gold) choose the most expensive generic item in the specified size. If the specified size is not available, choose the nearest larger size. If a larger size is not available, choose the nearest smaller size. Note the generic name in the "Your brand" and the size in the "Your size" columns.
3. Loose produce: choose the usual cheapest price per kg of the variety not on special. If the only variety available is on special, record the special price and note in comments column.
4. Peanuts: choose the branded packet size closest to 250 g. If packaged, roasted, unsalted peanuts are not available, record the price of the loose 'bulk scoop & weigh' roasted, unsalted peanuts per 100 g.
5. Check all data are collected and recorded as above, before leaving store.

Figure S1

Healthy Diets ASAP Protocol | The University of Queensland Australia – Create Change | Medical Research Future Fund | refresh – Centre of Research Excellence Food Retail Environments for Health | The Australian Prevention Partnership Centre | INFORMAS – Benchmarking food environments

Store name ________________ Store Location: _________________ Date: ___________ Collector: ________

Food	Specific brand	Your brand	Specific size	Your size	Your cost	Comments
Fresh Fruit						
Apples, red, loose			per kg			
Bananas, cavendish, loose			per kg			
Orange, loose			per kg			
Fresh Vegetables						
White potato, loose, brushed/washed			per kg			
Broccoli, loose			per kg			
Cabbage, white, ½ cabbage (or per kg)			½ cabbage or per kg			
Lettuce, iceberg, whole			Whole			
Carrot, loose			per kg			
Pumpkin, Jap, Kent, or Butternut			per kg			
Brown onion, loose			per kg			
Tomato, loose (not vine-ripened)			per kg			
Tinned Foods						
Tinned sweet corn, kernels, no added salt	Edgell		420g			
Tinned 4 bean mix	Edgell		420g			
Tinned tomatoes, diced/chopped in tomato juice	Ardmona		400g			
Fruit salad, canned/jar in juice	Goulburn Valley		700g			
Tinned steak & vegetables	Harvest		425g			
Tinned baked beans, in tomato sauce	Heinz		420g			
Tinned chicken & vegetable soup, ready to eat	Campbell's Country Ladle		505g			
Tuna, canned in vegetable oil, unflavoured	John West, Greenseas or Sirena		185g			
Pantry Food						
Wholemeal Bread	Tip Top Sunblest		700g			
White Bread	Tip Top Sunblest		700g			

Muffin, commercial, un-iced, any flavour, single or multipack	Supermarket		Record weight			
Rolled oats, whole, Traditional (not quick oats)	Uncle Toby's		1kg			
Cornflakes	Kellogg's		725g			
Weet-bix	Sanitarium		375g			
Spaghetti (white)	San Remo		500g			
White rice, medium grain	SunRice		1kg			
2 Minute noodles, chicken, single or 5/6 pack	Maggi or Fantastic		Record weight			
White Sugar	CSR		2kg			
Food	Specific brand	Your brand	Specific size	Your size	Your cost	Comments
Cream-filled biscuit	Arnott's Monte-Carlo		250g			
Chewy Choc Chip Muesli Bar	Uncle Toby's		185g			
Water Crackers, plain	Arnott's		125g			
Savoury flavoured biscuits	Arnott's BBQ Shapes		175g			
Peanuts – roasted, unsalted peanuts	Cheapest branded		250g			
Mixed nuts, (incl. peanut), salted	Nobby's		375g			
Mint confectionary	Allen's Minties		150g			
Dairy milk chocolate, block	Cadbury		200g			
Chips/crisps, original, salted	Smith's or Thins		170g			
French Dressing, regular fat	Praise		330mL			
Tomato sauce, regular (not ketchup)	Heinz Big Red or Masterfoods		500mL			
Sunflower oil	Crisco		750mL			
Olive oil, Traditional (not extra virgin)	Moro		1 Litre			
Meats						
Lean/4 star beef mince (not heart smart)	Pre-pack(not vacuum)		per kg			
Lamb loin chops	Pre-pack		per kg			
Beef rump steak	Pre-pack		per kg			
Beef Sausages, 6-8 pre-pack	Supermarket		per kg			
Refrigerated Items						
Cheddar cheese, regular fat	Cheer (Coon)		250g			
Cheddar cheese, reduced fat	Cheer (Coon)		250g			
Butter, original, salted (foil pack)	Western Star		250g			
Canola Margarine, regular fat	Meadow Lea		500g			

Full cream milk, fresh	Paul's or Dairy Farmers		2L			
Reduced fat milk, fresh (not skim)	Paul's Trim or Dairy Farmers Lite		2L			
Chocolate Milk, regular fat	Breaka, Big M, Oak or Paul's		600mL			
Orange Juice, Australian Grown (Fresh, chilled)	Berri		2L			
Plain Yoghurt, natural, Greek, regular fat (~4% fat)	Jalna		1kg			
Yoghurt, vanilla/flavoured, reduced fat (~1% fat)	Jalna		1kg			
Leg Ham, pre-pack	Don's		250g			
Food	Specific brand	Your brand	Specific size	Your size	Your cost	Comments
Eggs, dozen, Free Range	Sunnyqueen Farms		700g			
Drinks						
Bottled water, still	Mt Franklin		600mL			
Soft drink, Cola	Coca Cola		1.25L			
Diet soft drink, Cola	Coca Cola		1.25L			
Frozen Foods						
Frozen mixed vegetables	Heinz, Birdseye or McCain		500g			
Frozen peas	Edgell, Birdseye or McCain		500g			
Beef lasagne, frozen	McCain		400g			
White crumbed fish fillet, frozen	Birds Eye		425g			
Vanilla Ice cream, regular fat	Nestle Peters Original		2L			
Other Items						
Whole Barbeque Chicken, cooked - Large/ Family	Supermarket		Whole			
Pre-made Sandwich (Preferably chicken & salad on wholemeal bread)	Supermarket or, if unavailable, at closest garage/service station		2sl bread + filling (triangle pre-pack)			

Items from other stores:

**Food**	**Store**	**Your store**	**Specific size**	**Your size**	**Your cost**	**Comments**
Cooked hot potato chips	Independent Fish & Chip shop		1 serve			
Beef hamburger (Big Mac)	McDonald's		1 burger			
Beef Pie, single serve, full pastry	Independent Bakery		1 pie			
Supreme Pizza, thin base	Pizza Hut		1 large pizza			

Liquor Store Name: ______________________

**Food**	**Specific brand**	**Your brand**	**Specific size**	**Your size**	**Your cost**	**Comments**
Beer	VB		6 x 375mL			
Sparkling white wine	Yellow		750mL			
Whisky	Johnny Walker Red Label		700mL			
Red wine	Penfolds Koonungara Hill Shiraz		750mL			

Healthy Diets ASAP (Australian Standardised Affordability and Price) paper data collection form [12]

Table S3. *Welfare and low indicative disposable income calculations updated April 2024 [12]*

**Low indicative disposable income – Single parent family (Adult female, 14yr boy, 8yr girl)**		
**Assumptions**	• The adult female works on a casual basis at national minimum wage ($26.71/hr($23.23/hr + 15% casual loading)) for 25 hours a week for 39 weeks per year (not during school holidays)<br>• The adult female does not receive child support from the children's father<br>• Both children attend school and are fully immunised<br>• None of the family are disabled<br>• The family does not have savings or investments<br>• The family is privately renting their home at $450/week	Amounts per fortnight April 2024
**INCOME (FORTNIGHTLY)**		
Paid employment – adult female	$26.71/h/25h per week/39 weeks	$1,001.63
Parenting payment	$685.14/fortnight	$685.14
Family tax benefit A fortnightly payment	$490.84/fortnight	$490.84
Family tax benefit A annual supplement	$879.65/child/year	$67.67
Family tax benefit B fortnightly payment	$126.56/fortnight	$126.56
Family tax benefit B annual supplement	$430.70/family/year	$16.57
Rent assistance	221.20/fortnight	$221.20
INCOME TAX PAID	(Tax + medicare levy) – low income tax offset	$172.29
**TOTAL FORTNIGHTLY INCOME**		**$2,436.81**
	Work income	$26,042.25
	Parenting income	$17,813.64
	Total taxable income p.a	$43,855.89
	Tax payable p.a	$4,874.64
	LITO	$382.21
**Welfare dependant income - Single parent family (Adult female, 14yr boy, 8yr girl)**		
**Assumptions**	• The adult female is unemployed and looking for work<br>• The adult female does not receive child support from the children's father<br>• Both children attend school and are fully immunised<br>• None of the family are disabled<br>• The family does not have savings or investments<br>• The family is privately renting their home at $450/week	Amounts per fortnight April 2024
**INCOME (FORTNIGHTLY)**		
Parenting payment	$999.70/fortnight	$999.70
Family tax benefit A fortnightly payment	$490.84/fortnight	$490.84
Family tax benefit A annual supplement	$879.65/child/year	$67.67
Family tax benefit B fortnightly payment	$126.56/fortnight	$126.56
Family tax benefit B annual supplement	$430.70/family/year	$16.57
Rent assistance	$221.20/fortnight	$210.20
INCOME TAX PAID	(Tax + medicare levy) – low income tax offset	$ NIL

**TOTAL FORTNIGHTLY INCOME**		**$1,922.53**
**Low indicative disposable income – Family of four (Adult male, adult female, 14yr boy, 8yr girl)**		
**Assumptions**	• The adult male works on a permanent basis at national minimum wage for 38 hours a week ($23.23/hr)<br>• The adult female works on a part-time basis at national minimum wage ($23.23/hr) for 6 hours a week<br>• Both children attend school and are fully immunised<br>• None of the family are disabled<br>• The family has some emergency savings that earn negligible interest<br>• The family is privately renting a 3 bedroom house at $450/week	Amounts per fortnight April 2024
**INCOME (FORTNIGHTLY)**		
Paid employment – Adult female	$23.23/hr for 6h/week	$1,765.48
Paid employment – Adult male	$23.23/h for 38/h/week	$278.76
Parenting payment	$387.40/fortnight	$387.40
Family tax benefit A fortnightly payment	$490.84/fortnight	$490.84
Family tax benefit A annual supplement	$879.56/child/year	$67.67
Family tax benefit B fortnightly payment	$43.54/fortnight	$43.54
Family tax benefit B annual supplement	$430.7/family/year	$16.57
Rent assistance	$221.20/fortnight	$221.20
INCOME TAX PAID	(Tax + medicare levy) – LITO	$230.45
**FORTNIGHTLY INCOME TOTAL**		**$3,041.00**
	Male income p.a	$45,902.48
	Female income p.a	$7,247.76
	Female parenting payment p.a	$10,072.40
	Female total income p.a	$17,320.16
	Male tax payable p.a	$5.385.15
	Male LITO	$311.46
	Female tax payable p.a	$0
	Female LITO	$0
	Medicare levy	$918.04
**Welfare dependant income - Family of four (Adult male, adult female, 14yr boy, 8yr girl)**		
**Assumptions**	• The adult male is unemployed<br>• The adult female is unemployed<br>• Both children attend school and are fully immunised<br>• None of the family are disabled<br>• The family has some emergency savings that earn negligible interest<br>• The family is privately renting a 3 bedroom house at $450/week	Amounts per fortnight April 2024
**INCOME (FORTNIGHTLY)**		
Jobseeker payment	$706.20/fortnight	$706.20

Parenting payment	$706.20/fortnight	$706.20
Family tax benefit A fortnightly payment	$490.84/fortnight	$490.84
Family tax benefit A annual supplement	$879.65/child/year	$67.67
Family tax benefit B fortnightly payment	$35.56/fortnight	$35.56
Family tax benefit B annual supplement	$430.70/family/year	$16.57
Rent assistance	$221.20/fortnight	$221.20
INCOME TAX PAID	(Tax + medicare levy) – Low income tax offset	$0
**FORTNIGHTLY INCOME TOTAL**		**$2,244.23**
	Income p.a	$18,361.20
**Low indicative disposable income – Single adult male**		
**Assumptions**	• Works on a permanent basis at national minimum wage for 38 hours a week ($23.23/hr)<br>• Is not studying/training<br>• Is not disabled<br>• Has no dependent children<br>• Does not have savings or investments<br>• Is renting a room in 3 bedroom house at $150/week ($450/3)	Amounts per fortnight April 2024
**INCOME (FORTNIGHTLY)**		
Paid employment – Adult male	$23.23/hr for 38h/week	$1,765.48
INCOME TAX PAID	(Tax + medicare levy) – Low income tax offset	$230.40
**FORTNIGHTLY INCOME TOTAL**		**$1535.03**
	Income p.a	$45,902.48
	Tax payable p.a	$5,385.15
	LITO	$311.46
	Medicare levy	$918.04
**Welfare dependant income – Single adult male**		
**Assumptions**	• Has no paid employment but is looking for work<br>• Is not studying/training<br>• Is not disabled<br>• Has no dependent children<br>• Does not have savings or investments<br>• Is renting a room in 3 bedroom house at $150/week ($450/3)	Amounts per fortnight April 2024
**INCOME (FORTNIGHTLY)**		
Jobseeker payment	$762.70/fortnight	$762.70
Rent assistance	$115.60/fortnight	$115.60
Total clean energy supplement	$8.80/fortnight	$8.80
INCOME TAX PAID	(Tax + medicare levy) – Low income tax offset	$0
**FORTNIGHTLY INCOME TOTAL**		**$887.00**
	Income p.a	$19,830.20

International Journal of
Environmental Research and Public Health

MDPI

Table S4. *Summary outcome for habitual and recommended diets for each household ± SD and proportion of total cost (%) for each food group and discretionary foods for both diets (rounded to nearest whole number/ two decimal places) [12]*

	**Single parent family**				**Family of four**				**Single male household**			
	**Recommended**		**Habitual**		**Recommended**		**Habitual**		**Recommended**		**Habitual**	
**Food/food group**	**Mean cost ± SD ($AUD)**	**Share of total (%)**	**Mean cost ± SD ($AUD)**	**Share of total (%)**	**Mean cost ± SD ($AUD)**	**Share of total (%)**	**Mean cost ± SD ($AUD)**	**Share of total (%)**	**Mean cost ± SD ($AUD)**	**Share of total (%)**	**Mean cost ± SD ($AUD)**	**Share of total (%)**
Water, bottled	12.91 ± 1.08	**2**	12.91 ± 1.08	**2**	20.88 ± 1.74	**3**	20.88 ± 1.74	**2**	7.97 ± 0.66	**4**	7.97 ± 0.66	**3**
Fruit	73.65 ± 5.64	**13**	47.82 ± 2.56	**8**	99.04 ± 7.58	**13**	64.17 ± 3.33	**7**	25.39 ± 1.94	**12**	16.35 ± 0.78	**5**
Vegetables and legumes	102.41 ± 2.57	**18**	35.65 ± 0.98	**6**	142.41 ± 3.51	**18**	56.85 ± 1.54	**6**	40.00 ± 0.95	**19**	21.19 ± 0.57	**6**
Grain foods	100.06 ± 3.19	**18**	41.35 ± 1.09	**7**	134.69 ± 4.27	**17**	57.90 ± 1.47	**6**	34.63 ±1.08	**17**	16.55 ± 0.39	**5**
Lean meats and alternatives	167.07 ± 7.01	**29**	72.76 ± 3.38	**12**	228.04 ± 9.68	**29**	117.91 ± 4.48	**12**	60.96 ± 2.69	**29**	45.19 ± 1.12	**14**
Dairy and alternatives	104.59 ± 2.45	**18**	43.92 ± 1.57	**7**	136.96 ± 3.18	**18**	61.59 ± 2.16	**6**	32.37 ± 0.73	**16**	17.69 ± 0.59	**5**
Unsaturated fats	7.73 ± 0.38	**1**	1.41 ± 0.01	**<1**	14.06 ± 0.69	**2**	1.94 ± 0.02	**<1**	6.34 ± 0.31	**3**	0.53 ± 0.01	**<1**
Diet soft drink			4.49 ± 0.25	**1**			7.56 ± 0.42	**1**			3.07 ± 0.17	**1**
Soft drink			27.23 ± 1.51	**4**			37.98 ± 2.11	**4**			10.75 ± 0.60	**3**
Alcoholic drinks			29.88 ± 0.94	**5**			100.54 ± 1.31	**11**			70.73 ± 1.23	**22**
Takeaway foods			142.11 ± 20.29	**22**			198.32 ± 28.19	**21**			56.26 ± 7.96	**17**
Discretionary – other			173.02 ± 9.39	**27**			231.40 ± 12.24	**24**			58.42 ± 2.87	**18**
**TOTAL:**	568 ± 15	**99[a]**	633 ± 13	**101[a]**	776 ± 21	**100**	957 ± 18	**100**	208 ± 5	**100**	325 ± 5	**99[a]**
Healthy food/drink	568	**100%**	259.82	**41%**	776	**100%**	381.24	**40%**	208	**100%**	125.47	**39%**
Discretionary food/drink	Nil	**N/A**	376.73	**60%**	Nil	**N/A**	575.80	**60%**	Nil	**N/A**	199.23	**61%**

Percentages are rounded to the nearest whole number, totals are not always 100%

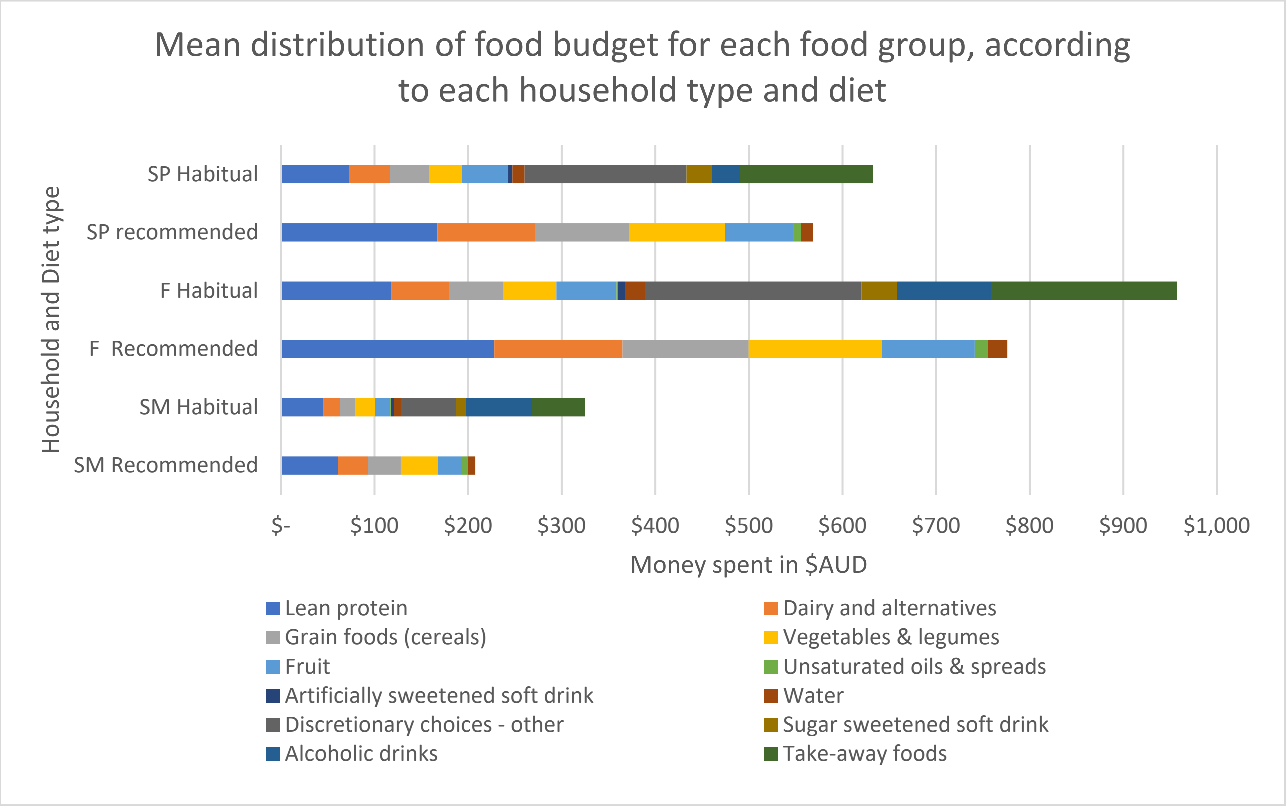

Figure S2. Proportion of food budget that would be spent on each food group for a family of four (F), single parent family (SP) and single male household (SM), according to habitual and recommended diets.

International Journal of
Environmental Research
and Public Health

12
